# ASO Author Reflections: The MelFo-Study, UK: Effects of a Reduced Frequency, Stage-Adjusted Follow-Up Schedule for Cutaneous Melanoma IB–IIC Patients After 3 Years

**DOI:** 10.1245/s10434-020-08763-5

**Published:** 2020-07-10

**Authors:** Marc Moncrieff, Beverly Underwood, Jennifer Garioch, Martin Heaton, Nakul Patel, Esther Bastiaannet, Josette Hoekstra-Weebers, Harald Hoekstra

**Affiliations:** 1 Department of Plastic and Reconstructive Surgery, Norfolk and Norwich University Hospital, Norwich, UK; 2 Norwich Medical School, University of East Anglia Faculty of Medicine and Health Sciences, Norwich, UK; 3 Department of Dermatology, Norfolk and Norwich University Hospital, Norwich, UK; 4 Department of Plastic Surgery, Norfolk and Norwich University Hospital NHS Foundation Trust, Norwich, UK; 5 Department of Surgery, Leiden University Medical Center, Leiden, The Netherlands; 6 Wenckebach Institute, Universitair Medisch Centrum Groningen, Groningen, The Netherlands; 7 Integraal Kankercentrum Nederland Locatie Groningen, Groningen, The Netherlands; 8 Department of Surgical Oncology, University Medical Center Groningen, Groningen, The Netherlands

## Past

The routine use of sentinel lymph node biopsy (SLNB) to accurately stage melanoma patients has been incorporated into most international melanoma guidelines. On average, over 80% of patients are sentinel node negative.^1^ However, these patients still require follow-up, since the risk of locoregional or distant spread, although comparatively low, remains a possibility. Approximately 90% of recurrences occur within 3 years of diagnosis for American Joint Committee on Cancer (AJCC) stage II melanomas, but for AJCC stage I melanomas; a significant proportion of recurrences occur after a substantial delay.^2^ Accordingly, it is challenging for national guidelines committees to devise simple follow-up schedules for melanoma patients.

## Present

The melanoma follow-up (MelFO) study is an international phase III randomized controlled trial where participants are randomized into two groups—one following the conventional schedule recommended in the UK National Institutes for Health and Care Excellence (NICE) melanoma guidelines,^3^ and one whose follow-up was an AJCC stage-adjusted reduced schedule. Importantly, the primary endpoint for this study is patients’ quality of life (QoL), although secondary endpoints include the usual standard outcomes data such as disease-specific and overall survival. We found that both cohorts expressed high satisfaction with their regimens (> 93%).^4^ Overall compliance with the follow-up schedules was high at the 1- and 3-year timepoints (68.5% and 66.5%, respectively). At 3 years, no significant group effect was found on any patient-reported outcomes measure scores, indicating no QoL difference between the follow-up protocols. The recurrence rate was identical (approximately 16%) and self-examination was the main method of detection for both groups (approximately 70%). Melanoma-specific survival was also identical.

## Future

These reflections were written at a time when most modern healthcare services have been disrupted beyond recognition during the COVID-19 pandemic. It is likely that many services will be rationalized in the future, once the pandemic subsides. Accordingly, evidenced-based follow-up regimens will be needed to ensure that the appropriate balance is struck between diagnosing early the few recurrences in low-risk groups such as these and not overburdening both patients and society alike.

The UK MelFO study has shown that 3 years after staging with a negative SLNB, AJCC stage IB–IIC cutaneous melanoma patients assigned to the prescribed reduced stage-adjusted follow-up schedule reported no difference in levels of anxiety, cancer worry, and mental health-related QoL when compared with those of patients assigned to the follow-up schedule as currently advised in the UK NICE melanoma guideline. These results mirror the findings of the Dutch group who recently reported very similar results using the same protocol in 2019.^5^ We anticipate reporting the combined data for the final outcome of the study at the end of 2020, with adequate power to detect any difference in recurrence rates. It is our hope that the results will assist future national guideline committees designing protocols for the follow-up of cutaneous melanoma patients.
